# Exploiting Sentinel-5P TROPOMI and Ground Sensor Data for the Detection of Volcanic SO_2_ Plumes and Activity in 2018–2021 at Stromboli, Italy

**DOI:** 10.3390/s21216991

**Published:** 2021-10-21

**Authors:** Alessandra Cofano, Francesca Cigna, Luigi Santamaria Amato, Mario Siciliani de Cumis, Deodato Tapete

**Affiliations:** 1 Italian Space Agency (ASI), Via del Politecnico s.n.c., 00133 Rome, Italy; alessandra.cofano@inaf.it (A.C.); deodato.tapete@asi.it (D.T.); 2 Mathematics Department, University of Rome Tor Vergata, Via della Ricerca Scientifica 1, 00133 Rome, Italy; 3 Institute for Space Astrophysics and Planetology (IAPS), National Institute for Astrophysics (INAF), Via del Fosso del Cavaliere 100, 00133 Rome, Italy; 4 Institute of Atmospheric Sciences and Climate (ISAC), National Research Council (CNR), Via del Fosso del Cavaliere 100, 00133 Rome, Italy; 5 Italian Space Agency (ASI), Località Terlecchia s.n.c., 75100 Matera, Italy; luigi.santamaria@asi.it (L.S.A.); mario.sicilianidecumis@asi.it (M.S.d.C.)

**Keywords:** Sentinel-5P, TROPOMI, ultraviolet, sulfur dioxide, SO_2_ plume, degassing, volcanic activity, Stromboli

## Abstract

Sulfur dioxide (SO_2_) degassing at Strombolian volcanoes is directly associated with magmatic activity, thus its monitoring can inform about the style and intensity of eruptions. The Stromboli volcano in southern Italy is used as a test case to demonstrate that the TROPOspheric Monitoring Instrument (TROPOMI) onboard the Copernicus Sentinel-5 Precursor (Sentinel-5P) satellite has the suitable spatial resolution and sensitivity to carry out local-scale SO_2_ monitoring of relatively small-size, nearly point-wise volcanic sources, and distinguish periods of different activity intensity. The entire dataset consisting of TROPOMI Level 2 SO_2_ geophysical products from UV sensor data collected over Stromboli from 6 May 2018 to 31 May 2021 is processed with purposely adapted Python scripts. A methodological workflow is developed to encompass the extraction of total SO_2_ Vertical Column Density (VCD) at given coordinates (including conditional VCD for three different hypothetical peaks at 0–1, 7 and 15 km), as well as filtering by quality in compliance with the Sentinel-5P Validation Team’s recommendations. The comparison of total SO_2_ VCD time series for the main crater and across different averaging windows (3 × 3, 5 × 5 and 4 × 2) proves the correctness of the adopted spatial sampling criterion, and practical recommendations are proposed for further implementation in similar volcanic environments. An approach for detecting SO_2_ VCD peaks at the volcano is trialed, and the detections are compared with the level of SO_2_ flux measured at ground-based instrumentation. SO_2_ time series analysis is complemented with information provided by contextual Sentinel-2 multispectral (in the visible, near and short-wave infrared) and Suomi NPP VIIRS observations. The aim is to correctly interpret SO_2_ total VCD peaks when they either (i) coincide with medium to very high SO_2_ emissions as measured in situ and known from volcanological observatory bulletins, or (ii) occur outside periods of significant emissions despite signs of activity visible in Sentinel-2 data. Finally, SO_2_ VCD peaks in the time series are further investigated through daily time lapses during the paroxysms in July–August 2019, major explosions in August 2020 and a more recent period of activity in May 2021. Hourly wind records from ECMWF Reanalysis v5 (ERA5) data are used to identify local wind direction and SO_2_ plume drift during the time lapses. The proposed analysis approach is successful in showing the SO_2_ degassing associated with these events, and warning whenever the SO_2_ VCD at Stromboli may be overestimated due to clustering with the plume of the Mount Etna volcano.

## 1. Introduction

A largely exploited proxy to study volcanic activity and its associated hazards is sulfur dioxide (SO_2_). The flux of SO_2_ is often considered as a precursor to eruptions and a marker of major volcanic processes (e.g., [1,2,3]). Further information is given by SO_2_ if considered in relation to other gases, so as to define a comprehensive inventory of gas emissions from volcanoes, providing additional constraints to volcanic activity and degassing [4,5,6].

Following water vapor and carbon dioxide (CO_2_), SO_2_ is among the most abundant gases involved in volcanic activity. At the same time, it is relatively easy to detect in areas isolated from inhabited centers and anthropogenic sources. This is also due to its generally low background concentration in the atmosphere [6].

SO_2_ degassing is directly associated with magmatic activity, thus informing on the style and intensity of the eruption, which are essential to study volcanic hazard and risk [6,7]. Therefore, assessing SO_2_ emissions and their temporal evolution through time series analysis can be very useful for volcanic monitoring purposes to better understand hazard and try to reduce the risk associated with volcanic activity. With this goal in mind, this paper aims to exploit the satellite dataset of atmospheric SO_2_ monitoring records at an unprecedented spatial resolution and sensitivity provided by the Sentinel-5 Precursor (Sentinel-5P) atmospheric chemistry mission of the Copernicus Programme.

Launched on 13 October 2017 as a precursor to Sentinel-4 and Sentinel-5, Sentinel-5P was developed to bridge the data gaps between current and future missions. Compared to those of other nadir sensors onboard atmosphere monitoring missions, such as the Ozone Monitoring Instrument (OMI) of NASA’s EOS/Chem-1 Aura mission [8] and the Infrared Atmospheric Sounding Interferometer (IASI) of the ESA MetOp mission [9,10], the push-broom nadir imaging spectrometer onboard Sentinel-5P—namely TROPOspheric Monitoring Instrument (TROPOMI)—has an improved spatial resolution (i.e., 3.5 km × 7 to 5.5 km), better detection limit to SO_2_ emissions (by a factor of 4; [11]) and sensitivity characteristics.

Such technical improvements enable higher precision measurements of volcanic concentrations of SO_2_, and consequently, more pixels of the satellite images cover the volcanic plume, the SO_2_ signal is less diluted and higher concentrations are detected. At the same time, different plumes, from either the same or different emitters, can be distinguished [11,12]. The detectability of SO_2_ emissions is also not limited to major explosive eruptions, strongly degassing and/or high elevation volcanoes, but can be extended to weaker SO_2_ degassing plumes [11]. Moreover, TROPOMI’s improved characteristics pave the way for trialing volcano studies reaching up to the local scale to evaluate the potential to monitor relatively small sites and emitters, trying to understand how identifiable and measurable (or not) SO_2_ emissions are from a point-wise volcanic source.

**Figure 1:**
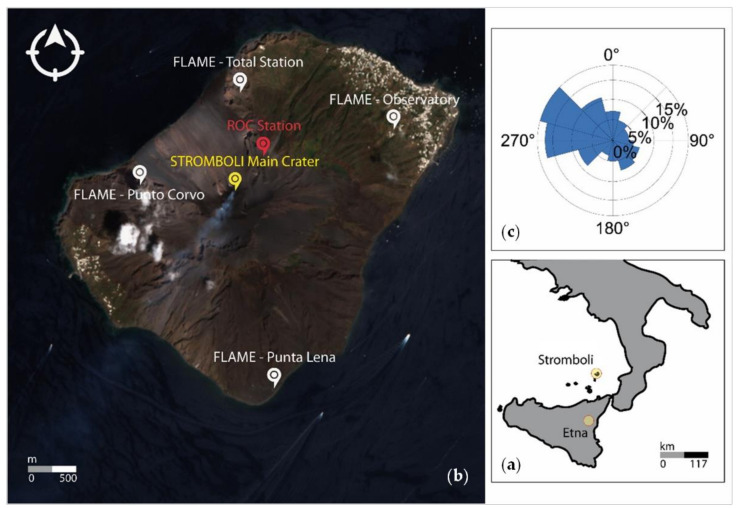
(**a**) Location of the Stromboli volcano in southern Italy and (**b**) satellite overview of the volcano edifice from Copernicus Sentinel-2 optical imagery acquired on 11 August 2019. The placemarks indicate: the main crater (38.79° N, 15.21° E) and five ground sensors belonging to the FLAME (white) network and ROC (red) station, respectively [14,15]. (**c**) Wind frequency rose at the 100 m reference height above ground level, estimated using measurements from the Energy Sector Management Assistance Program (ESMAP) campaigns and ECMWF Reanalysis v5 (ERA5) long-term reference data (source: https://globalwindatlas.info/; accessed on 1 October 2021).

To achieve this demonstration, this study focuses on the Stromboli volcano in southern Italy (Figure 1a). Its constant activity, characterized by persistent degassing [6,13], makes Stromboli an ideal test case for such an assessment, with a view to distinguish periods at different activity intensities and recognize the phenomenology associated with a transition between two distinct phases. Furthermore, Stromboli is the volcano from which the whole category of volcanoes characterized by violent and explosive eruptions is named. Therefore, it also serves as an ideal testing ground for applications to other Strombolian volcanoes. During such eruptions, continuous degassing occurs and may last even for several years, leading to gas emissions comparable to large eruptions, and a significant amount of magma is involved in this activity [6]. Often, explosions last a few minutes at most, and recur at various intervals. In particular, explosions at Stromboli last 15 s (i.e., ~13 explosions in an hour) and SO_2_ emission rates are in the order of ~730 t/d [6,13]. Such phenomenology requires an observational capability of not only suitable spatial resolution, but also high temporal frequency of data collection that, among the current space sensors, Sentinel-5P is able to provide.

It is beyond the scope of this work to compare Sentinel-5P data acquired over Stromboli with observations from previous satellite missions. The goal is instead to extract the time series of SO_2_ column density and make a comparison with information on activity based on ground sensor data and multispectral imagery from other satellite missions. This is also in preparation for future integration with aerial sensor data (by airplane or drone), which could bridge the interpretation of satellite data with ground observations.

An increasing number of volcanic studies have exploited TROPOMI data in the last three years [11,12,16,17,18,19,20,21,22,23], following the recent launch of the mission. Compared to this literature, the present work aims to:

(1) Discuss and address the technical issues involved in TROPOMI SO_2_ data extraction, filtering by quality, outlier removal and time series generation to monitor small-size Strombolian volcanoes. To this scope, TROPOMI SO_2_ data processing is based on purposely adapted Python scripts;
(2) Demonstrate a methodological and practical workflow for time series analysis enabling the spatio-temporal investigation of the key parameter of SO_2_ column density and its trend and variations, in order to better understand the activity of the volcano during and in between different eruptions. The demonstration is run with regard to events that occurred in 2018–2021 and also encompasses integration with ground data from permanent monitoring networks deployed across the volcano edifice, as well as contextual Sentinel-2 multispectral and Suomi NPP VIIRS observations.

## 2. Study Area and Recent Events

The Stromboli volcano is an island belonging to the Archipelago of the Aeolian islands, an active volcanic arc located to the north of Sicily (Italy), in the southern Tyrrhenian Sea (Figure 1a). The whole island has a width varying from 2.5 to 3.5 km and a length of 4 km (Figure 1b) that, for the purpose of this study, make the volcano size comparable with a TROPOMI single pixel (see Section 3.1). Its activity occurs at an altitude of 750 m a.s.l. from the various eruptive vents that line up in the NE–SW direction [24,25,26]. In addition to the normal continuous degassing activity, sequences of explosions of higher energy occur, called paroxysms, with the ejection of lithic blocks of various sizes onto the volcano flanks, down to lower elevations. These events may impact inhabited areas, can be accompanied by earthquakes and are typically followed by the actual eruption phase [24,25,26].

Periods of inactivity are quite rare for Stromboli, and this makes monitoring this volcano even more important to prevent impacts on local inhabitants and tourists. In recent years, an unexpected eruption occurred on 3–4 July 2019 and caused one death [27]. This event was part of one of the most recent paroxysmal periods. The paroxysm started on 3 July 2019, with strong explosions, a raising column of smoke and ash, lava and pyroclastic flows running along the “Sciara del Fuoco” (Stream of Fire) in the north-western flank of the volcano [26,28,29]. After a period of more moderate eruptions lasting almost two months, on 28 August 2019 a further paroxysm occurred, and was followed by two new explosions of slightly lower intensity between 29 and 30 August 2019. On 19 July 2020, another weaker paroxysm happened. Finally, on 19 May 2021, the last paroxysm of a further lower intensity but involving a major explosive sequence was detected [28,29,30]. All these events are of particular importance as they fall within the time period investigated in this paper with the Sentinel-5P dataset.

The present study also focuses on major explosions. Although they have lower intensity than paroxysms, their activity is typically higher than ordinary (or persistent). The most recent occurred on 13 August, 16–21 November and 6 December 2020, and on 14–18 January and 1 March 2021 [28,29,31,32]. Finally, another interesting period of activity was recorded between March and April 2020, with less intense phenomena than the abovementioned periods. A comparison with the latter would be worthwhile, since this eruption was not characterized by strong emissions of SO_2_, but by pyroclastic density currents and overflows [33].

A further aspect that has been accounted for in this study is the geographical proximity of Stromboli to the Mount Etna volcano (~120 km) (Figure 1a), with specific regard to how this distance factor (combined with other environmental conditions) may affect the reliability of the analysis in the framework of the satellite SO_2_ monitoring approach based on Sentinel-5P observations (see Section 4). According to the measurements from the Energy Sector Management Assistance Program (ESMAP) campaigns and ECMWF Reanalysis v5 (ERA5) data, the prevalent wind direction at Stromboli is W–NW (Figure 1c), and the same direction is most frequently recorded over Mount Etna. However, this is an indicative long-term reference only, as it accounts for the whole last decade. Hourly records of wind speed and direction at the two volcanoes on specific days can help to contextualize one of the key environmental factors that may cause plume drift and potential overlap between the two volcanoes.

## 3. Materials and Methods

### 3.1. Sentinel-5P TROPOMI SO_2_ Data

The main input dataset of this study is the whole record of Sentinel-5P TROPOMI data acquired over Stromboli, since the beginning of routine operations for the mission, after its 6-month-long commissioning phase. In particular, the TROPOMI time series for Stromboli starts on 6 May 2018, and the analyzed data cover three full years of observations, until 31 May 2021.

Sentinel-5P is a sun-synchronous, quasi-polar low-Earth orbit (824 km) satellite, allowing daily global coverage. An orbital cycle lasts 16 days, i.e., 14 orbits per day, and 227 orbits per cycle on average [34]. However, a daily or sub-daily revisit could be achieved owing to the 108° across-track field-of-view of TROPOMI and the possibility to cover the site with multiple tracks [11].

TROPOMI acquires data in four different spectral regions (ultraviolet, visible, near and short-wave infrared) that allow the observation of SO_2_, among many other gases [34]. TROPOMI’s detection limit to SO_2_ emissions is a factor of 4 better than OMI [11]. At nearly equal footprint (diameter of 12 km), the sensitivity of TROPOMI to SO_2_ variations is higher than IASI’s [9].

Compared to OMI’s minimum pixel size of 13 km × 24 km, TROPOMI definitely augments the level of spatial resolution over Stromboli. The nominal pixel size near the nadir is 3.5 km (across-track) × 7 km (along-track) for the data acquired until August 2019, then it was improved to 3.5 km × 5.5 km for more recent imagery [35]. It is worth mentioning that this size of the pixel may vary significantly across-track, especially when approaching the margins of the swath, where pixels may stretch along several kilometers in the ~east–west direction and hence become not suitable for further analysis at the local scale. Swath edge pixels are also characterized by higher standard errors [18]. TROPOMI products where Stromboli was imaged very close to the swath edge were therefore excluded from the present analysis. Nevertheless, the daily revisit in the analyzed set of data was preserved, thanks to the availability in the catalogue of more than one scene per day where the island was imaged, and among which the most suitable scenes could be selected for each date. Overall, a total of 1370 products were selected from the catalogue, downloaded and used for the analysis.

The investigation area also includes the whole Aeolian archipelago as well as north-eastern Sicily and the Mount Etna volcano to be able to identify the SO_2_ plumes of this active stratovolcano and how they may interfere with those of Stromboli. Considering Sentinel-5P’s orbit and its 2600 km swath width, the data for this area correspond approximately to the time slot 09:45–13:15 UTC.

The downloaded TROPOMI data are standard Level 2 (L2) SO_2_ geophysical data products in netCDF-4 format (.nc), made available via the European Space Agency (ESA) Copernicus Open Access Hub in the Sentinel-5P Pre-Operations Hub, and generated within the Copernicus ground system with a workflow developed by the Royal Belgian Institute for Space Aeronomy (BIRA-IASB) [35]. L2 products are obtained from Level 0 (L0) raw data, calibrated and georeferenced, and processed to Level 1 (L1b), i.e., radiance and irradiance. Finally, L2 products with SO_2_ concentrations are extracted from the ultraviolet (UV) spectrum using algorithms based on Differential Optical Absorption Spectroscopy (DOAS) and a combination of three fitting windows: 312–326, 325–335 or 360–390 nm [23]. For space observations, this method involves two steps. The absorption cross-sections are first adapted to the measured terrestrial luminosity spectrum (normalized with respect to the solar irradiance) to obtain the slant column density (SCD). Then, the SCD is converted into the SO_2_ vertical column density (VCD) using the air mass factor (derived from radiative transfer calculations), which accounts for changes in measurement sensitivity (e.g., due to clouds and aerosols, surface reflectivity, as well as best-guess SO_2_ vertical profiles) [23]. SO_2_ integrated column density values in mole concentrations (mol/m^2^) can be expressed in Dobson Units (DU), where 1 DU = 2.69 × 10^16^ molecules/cm^2^, and indicate the number of SO_2_ molecules in an atmospheric column per unit area.

The products used for the analysis were available from three different data streams, depending on the L2 processor version [35]: reprocessing (RPRO) stream for data acquired between May and November 2018; non-time critical or offline (OFFL) for data between December 2018 and May 2021; and near real-time (NRTI) for the product collected on 28 August 2019 (due to a shift observed in the geolocation of the corresponding OFFL product, making it not suitable for the following analysis).

### 3.2. Python Algorithm and Data Analysis

Data analysis was carried out using the Anaconda platform and Python programming language. The Sentinel-5P Python routines distributed by NASA [36] to extract information on other gases (e.g., “*read_tropomi_no2_ai_at_a_location.py*” for NO_2_ products) were purposely modified to read the SO_2_ products and adapt the script to this study.

In order to extract the full time series, the workflow of the original Python routines was automated for the 1370 L2 files to process. A dedicated step allowing the opening and writing of an Excel file was added, so that the output data were exported into a suitable format, ready for further analysis. Additional commands to extract the acquisition date and the representation with the relative error bar were also introduced. The “*sulfurdioxide_total_vertical_column*” value (i.e., the total atmospheric column between the surface and the top of the troposphere) and the respective errors originating from the spectral fit (i.e., the random “*sulfurdioxide_total_vertical_column_precision*” and systematic “*sulfurdioxide_total_vertical_column_trueness*” errors, i.e., precision and accuracy) were extracted from the data files. To read and convert 1370 products and their metadata, the script execution time lasted about 50 min.

**Figure 2:**
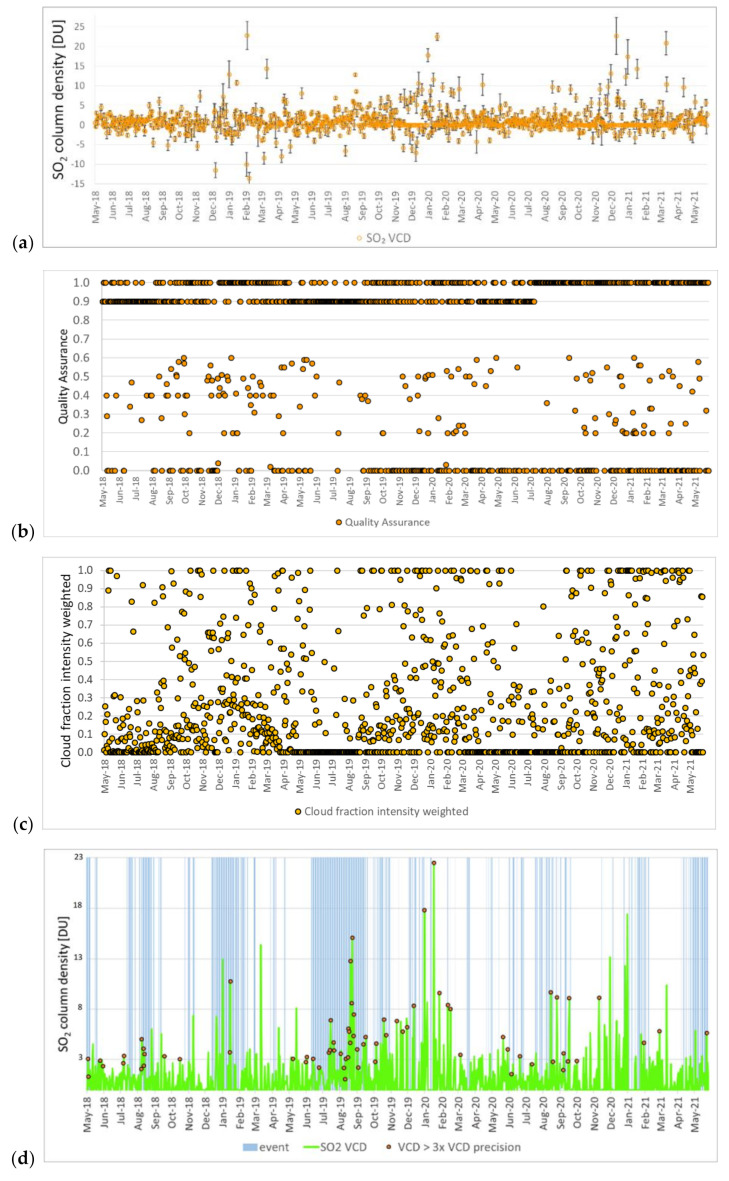

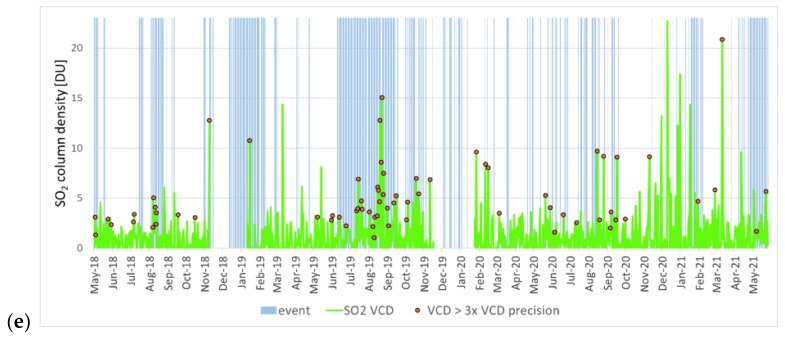
(**a**) TROPOMI sulfur dioxide (SO_2_) vertical column density (VCD) observations and respective error bars (standard deviation) in May 2018–May 2021 at Stromboli (38.79° N, 15.21° E). (**b**) Quality Assurance (QA) values and (**c**) “cloud flag” associated with SO_2_ data. (**d**) SO_2_ VCD observations after QA and (**e**) best quality data selection filtering, with indication of the periods of intense volcanic activity (indicated in the plot as “event”) as per the information derived from UNIFI’s daily bulletins based on the ROC ground sensor data (see red placemark in Figure 1b for location).

The script reads the input latitude and longitude (Stromboli’s main crater; 38.79° N, 15.21° E) and finds the closest pixel to the coordinates selected. Given that TROPOMI pixel size suits the extent of the Stromboli volcano (see Section 3.1), the use of a single pixel enables the sampling of the whole island. Figure 2a shows the full record of the total vertical column of SO_2_ over time at the main crater, with respective error bars. The total column density values exceed 10 DU (26.9 × 10^16^ molecules/cm^2^) on several dates across the three-year-long observation period. Errors associated with the column density values are on average ~1.5 DU (4.1 × 10^16^ molecules/cm^2^) across the whole time period, but generally lower than 1.2 DU (3.2 × 10^16^ molecules/cm^2^) for a large portion of the series, and above 3.0 DU (8.1 × 10^16^ molecules/cm^2^) only at a few dates.

In order to avoid misinterpretations of data quality, the Quality Assurance (QA) parameter associated with each product (Figure 2b) was used to filter out the data before further analysis. It is indicated by the “*qa_value*” flag, and provides a continuous value expressing a quality percentage: from 0, which indicates a processing error, to 1 (or 100%), which is the optimal value. The QA accounts for several quality parameters and factors, including the possible presence of clouds, snow or ice on the surface. In this quantity, there are also caveats related to the South Atlantic Anomaly, the sun glint or missing input data, which lower the QA further. In the current phase, the Mission Performance Centre (MPC) and the Sentinel-5P Validation Team (S5PVT) therefore recommend users to adopt a QA > 0.50, or even a more conservative thresholding with QA > 0.75, to avoid considering a large proportion of clouds and snow/ice-covered scenes. Given the position of the SO_2_ emitter of our study, that is, far enough from anthropogenic sources that may compromise the data quality and climatologically rarely exposed to snow and ice, the QA threshold was set at 0.50. By following this approach, a total of 498 low quality SO_2_ column values were filtered out, and therefore excluded from the following analysis.

For products processed using the first version of the SO_2_ processor, in addition to the QA filter, the MPC experts and the S5PVT [35] recommend to use the following additional filters for best quality data selection, namely: snow/ice flag (“*snow_ice_flag*”) < 0.5; total VCD (“*sulfurdioxide_total_vertical_column*”) > −0.001 mol./m^2^; total air mass factor for boundary layer polluted scenario (“*sulfurdioxide_total_air_mass_factor_polluted*”) > 0.1; effective radiometric cloud fraction from the Clouds-as-Reflecting-Boundaries model (“*cloud_fraction_crb*”) < 0.3; and solar zenith angle < 60°. Data filtering according to these more stringent criteria was therefore implemented for all products collected over Stromboli until 12 July 2020 (i.e., those processed with software versions v.1.1.5–1.1.8).

The cloud radiance fraction (“*cloud_fraction_intensity_weighted*”, i.e., “cloud flag”) was also extracted, and its values for the whole time series are shown in Figure 2c. This parameter is dimensionless and indicates the intensity-weighted cloud fraction (VCD clear sky vs. cloudy weighting factor), ranging between 0 (clear sky) and 1 (cloudy) [23]. After filtering, the cloud radiance fraction values during the May 2018–May 2021 period were mostly in the 0 to 0.2 range, with a limited number of dates when it was between 0.2 and 0.4, and only a few when it exceeded 0.4.

The time series after QA (Figure 2d) and further filtering includes 589 observations (Figure 2e). The VCD errors of the filtered dataset are on average ~2.1 DU (5.6 × 10^16^ molecules/cm^2^). In the same plot, the markers indicate when the observed SO_2_ column values were above three times their associated precision (i.e., VCD > 3× VCD precision), showing robust evidence of the occurrence of a peak, often associated with events detected by ground instrumentation (see Section 3.3). Comparison of the plots in Figure 2d,e allows for the identification of the loss in VCD records due to the use of the additional quality filters. Whilst on one hand these filters help to ensure the selection of best quality data, on the other hand, they might cause data loss in specific dates due to too stringent thresholds, or even in specific periods due to higher solar zenith angles (e.g., November to January at Stromboli’s latitudes).

It is worth noting that, since September 2019, when the along-track pixel width of TROPOMI was changed, the VCD values across the time series in Figure 2d appear to be generally higher than before. This is because by decreasing the pixel area (and therefore improving the resolution), the plume is less diluted and the local maxima are better resolved. This change is important for small volcanoes such as Stromboli, which may have concentrated plumes close to the emission source. More in general, this also matches with a consideration by Theys et al. [11], who made a comparison with the predecessor OMI.

A further modification to the original Python codes concerned the spatial scale of analysis, and the possibility to generate maps for only a subset of the SO_2_ product (instead of its whole extent). To this aim, the original code (*read_and_map_tropomi_no2_ai.py*) was edited to be able to input the location of the island of Stromboli, thus enabling the zoom onto the study area.

Finally, SO_2_ VCD values for three different peak altitudes above the surface, representing different altitude regimes (“*sulfurdioxide_total_vertical_column {1,7,15} km*”), were also extracted from the L2 data files. These are total SO_2_ columns considering 1-km-thick box profiles at ground level, and centered at 7 and 15 km a.s.l., respectively. Their time series were filtered by using the recommendation provided by the MPC experts and the S5PVT (i.e., solar zenith angle < 70°) [35]. Similarly to the total column, the estimates of random and systematic errors are provided for the SO_2_ columns for the three box profiles [35].

If both SO_2_ plume height and wind speed data were available at the location of the volcano and at the satellite overpass times (e.g., from ground-based instrumentation), an estimation of TROPOMI-based SO_2_ flux could also be attempted, as performed for instance by Theys et al. [11] using the cross-sectional flux method.

### 3.3. Ground-Based Sensor Data

The ground-based sensor data that provided information on volcanic activity were obtained from the FLux Automatic MEasurements (FLAME) and ROCcette site (ROC) stations (see their location in Figure 1b). These instrumental measurements were retrieved from volcanic activity bulletins published online by the National Institute of Geophysics and Volcanology (INGV) [37,38] and the Laboratory of Experimental Geophysics (LGS) at the Department of Earth Sciences of the University of Florence (UNIFI) [39,40], respectively. These research centers have deployed several sensor networks across the island to study the various phenomena related to Stromboli’s volcanic activity and, among the deployed networks, ground instrumentation to measure SO_2_ emissions.

The spectrometers located in each station measure the absorption of UV radiation of the volcanic plume. Columnar quantities of SO_2_ are detected and they are expressed in ppm·m (parts per million meters). To estimate the flow of SO_2_ emitted by the volcano, expressed in t/d (tons per day), the columnar quantity of SO_2_ is multiplied by the wind speed at that altitude (assuming that the movement speed of the gaseous mass is the same as that of the wind) [38,39].

Bulletin data are used in this study, primarily to distinguish periods of intense activity from periods with low emissions and tremors. The validation of satellite estimates by means of ground sensor data is beyond the scope of this paper. Therefore, the complete information provided by all the instruments deployed at the volcano was first considered, and finally the SO_2_ records were used to make a qualitative comparison only between the quantity of gas emitted on the ground compared to that observed by the satellite. The periods of more intense activity are highlighted in light blue (“events”) in the various time series graphs presented in this paper.

As discussed later in Section 4.2 and in the literature [6,13], the analysis of data from ground-based sensors does not always allow an association between strong SO_2_ emissions and intense Stromboli activity. For this reason, when identifying and cataloguing the events from the analysis of the bulletins (especially those published by UNIFI), the rationale was to consider “periods of activity”—those characterized by medium to very high SO_2_ emissions (as measured by the ground instruments). This is indicated on the UNIFI website, in particular on the daily charts for the ROC station.

Hourly records of wind direction and wind speed available from weather stations belonging to the World Meteorological Organization (WMO) network were accessed and also used to contextualize satellite observations. In particular, we used data from the 3 stations established at: (i) the town of Piscità in the eastern sector of the island (38.80° N, 15.23° E, 75 m elevation) for which, however, information on winds is not consistently available for the whole period covered by the TROPOMI dataset but only starting in early 2021, thus limiting the use of these data to specific dates only; (ii) the city airport of Catania (37.47° N, 15.05° E, 11 m), south of Mount Etna; and (iii) the island of Salina located 50 km south-west of Stromboli (38.58° N, 14.87° E, 46 m).

## 4. Results and Discussion

### 4.1. Spatial Averaging and Ratioing

The value of the total vertical column of SO_2_ at a single pixel may not be exhaustive to describe the investigated phenomenon, as SO_2_ emissions will likely extend beyond the size of the pixel. Therefore, a technical question to answer is whether expanding the coverage of the sampled area and considering other pixels over a N × M window is a suitable approach to provide robust metrics for the investigated site, and how many of such pixels should be considered.

**Figure 3:**
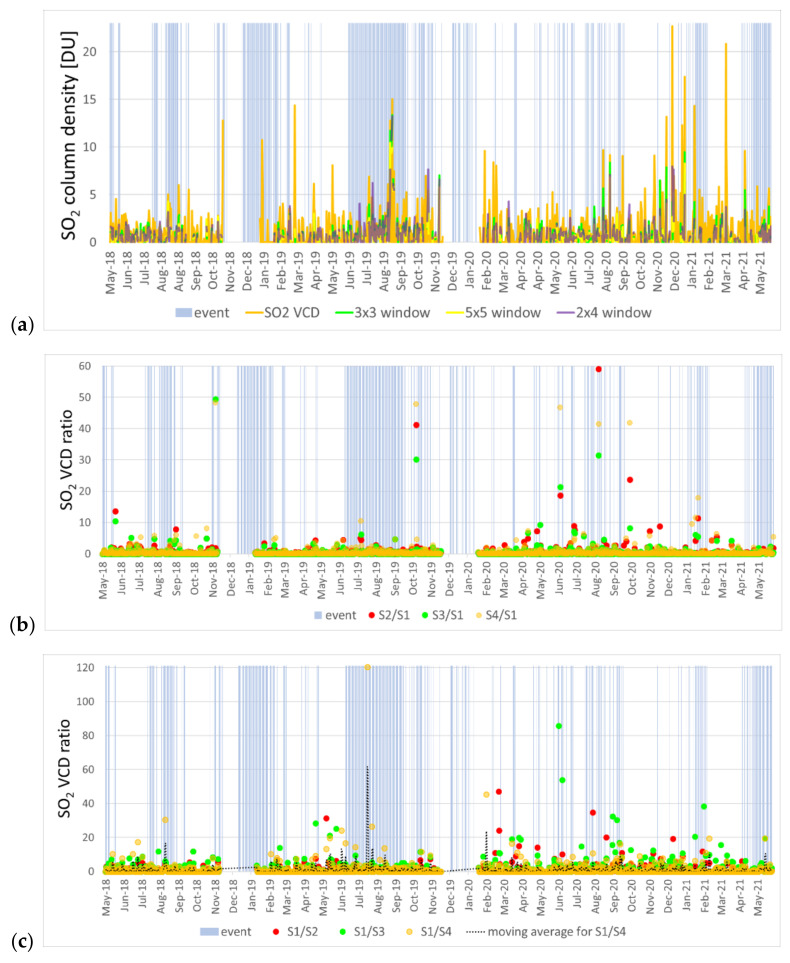
(**a**) Comparison of TROPOMI SO_2_ vertical column density (VCD) observations in May 2018–May 2021 for the main crater of Stromboli, by sampling at a single pixel centered at the crater (S1) and using three averaging windows (i.e., S2, S3 and S4; i.e., calculated over 3 × 3, 5 × 5 and 4 × 2 windows, respectively). Ratios between observations at the single pixel and within the averaging windows: (**b**) S2/S1, S3/S1 and S4/S1; and (**c**) S1/S2, S1/S3 and S1/S4.

To answer this question, SO_2_ average values over 3 × 3 and 5 × 5 pixels were first extracted, thus adopting symmetrical windows that provide 10.5 × 21 and 17.5 × 35 km^2^ (at nadir) coverage until August 2019, and 10.5 × 16.5 and 17.5 × 27.5 km^2^ after that date, respectively. The rectangular output areas, however, do not preserve the geometric proportions of the investigated area. Moreover, too large sizes produced by the 5 × 5 window may attenuate the SO_2_ emission of the volcano with surrounding pixels characterized by lower concentrations. Therefore, a 4 × 2 window was also considered, thus covering a square area of 14 × 14 km^2^ until August 2019, and 14 × 11 km^2^ after that date, respectively. Figure 3a illustrates the output SO_2_ column density time series for the single pixel at the main crater and the tested 3 × 3, 5 × 5 and 4 × 2 averages.

Peaks visible in the single pixel time series decrease rapidly with increasing window size. For instance, on 5 December 2020, a peak of ~13.2 DU (35.4 × 10^16^ molecules/cm^2^) is observed at a single pixel over the main crater, then a drop to ~7.9 DU (21.1 × 10^16^ molecules/cm^2^) is found using a 3 × 3 window, ~4.1 DU (11.0 × 10^16^ molecules/cm^2^) with a 5 × 5 window and ~4.4 DU (11.9 × 10^16^ molecules/cm^2^) with the adapted 4 × 2 window (Figure 3a). Since the peak refers to a point-wise emitter inside the single TROPOMI pixel, by increasing the spatial sampling window, the total averaged signal generally decreases. Vice versa, higher values observed over larger windows may indicate that strong emissions from other sources may have been included within the averaging window (e.g., a plume originating from Mount Etna). This comparison can also provide supporting evidence to verify whether some peaks recorded at a single SO_2_ pixel outside periods of volcanic activity are reliable or not.

The graph in Figure 3a shows that often the 4 × 2 series (in purple) agrees with the trend of the single pixel (in yellow). Sometimes there is a greater agreement with the 3 × 3 series but, when considering a greater area such as the 5 × 5 window, the risk of finding negative or low values increases, and the averaging smoothes the SO_2_ records. Therefore, the 4 × 2 averaging area generally might be considered a more reliable sampling window for this volcano.

In general, for each window, with the improvement of TROPOMI’s resolution since September 2019, a greater consistency has been achieved between the single pixel VCD and the different windows. Although the pixel shape has changed, the 4 × 2 window remains the most consistent with the single pixel VCD.

A peak ratioing analysis was carried out to further investigate the peak attenuation effect produced on the observed values of SO_2_ emissions by changing the window sizes and shape, and to better understand if even the single pixel can be sufficient to describe the SO_2_ trend for a relatively small volcano such as Stromboli. The heights of the peaks were compared by ratioing the average VCD value for the 3 × 3 (hereafter identified as S2), 5 × 5 (S3) and 4 × 2 (S4) windows by the VCD value at the single pixel (S1). Figure 3b shows the values of S2/S1, S3/S1 and S4/S1, while in Figure 3c the numerator and the denominator are inverted.

By carefully analyzing the values of peaks S1, S2, S3 and S4 (Figure 3a) and of the respective ratios, it appears that the highest ratio values in the first plot (Figure 3b) often correspond to peaks that are not particularly significant (and, in some cases, almost below a level that can be considered as “background noise”). On the other hand, in the presence of well-defined occurrences of high SO_2_ emissions across more pixels within the averaging window, the ratios are typically lower (equal to ~1), as the emissions are sensed not only at the single pixel but also across the windows, which may either encompass emissions from other sources (e.g., Mount Etna) or Stromboli itself, in the case of major events (if the SO_2_ plume extends over the whole averaging window).

The plot in Figure 3c might be more helpful to identify dates when SO_2_ vertical column density observations at the crater stand out significantly from those across the averaging windows, and thus support the detection of high emissions from the volcano. Table 1 summarizes the number of days when each ratio S1/S*n* (either S1/S2, S1/S2 or S1/S3) equals or exceeds a set threshold *R*, by distinguishing between days of low SO_2_ flux (<70 t/d) and days of medium to very high flux (≥70 t/d) as detected at the ground-based instrumentation at the ROC station. The statistics show that the S1/S2 ratio exceeds the threshold of 5 for a total of 38 days, including 9 days (i.e., 26% of the dates with available flux information at ROC) when ground instrumentation measured an SO_2_ flux of at least 70 t/d, 25 days when the flux was low and 4 days when no flux information was available at the station. Only during 14 days the ratio was above 10, with four (40%) of such occurrences corresponding with days of medium to very high flux at the ROC station, and six with low flux. By enlarging the averaging window to the 5 × 5 size, the ratios S1/S3 indicate that the threshold of 5 was exceeded on a total of 74 days, of which 18 (26%) were of medium to very high flux, and 52 of low flux. Most of these detections are observed after mid-2019. On the other hand, the higher threshold of 10 was exceeded on 29 days only, of which 6 (23%) were of medium to very high flux, and 20 of low flux.

Much more effective seems to be the exploitation of the 4 × 2 window size, for which the ratio S1/S4 was at least 5 on a total of 59 days, of which 21 (41%) corresponded with medium to very high flux at the ROC station. This is particularly apparent in the first half of the series, approximately until August 2019, by which most of these occurrences can be observed (Figure 3c). By increasing the threshold to 10, the number of detections drops to 22, though a much larger proportion of the days when flux data were available (50%) is found for days of medium to very high flux at the ROC station.

The temporal distribution of the good matches between higher ratio values and the days of SO_2_ flux ≥ 70 t/d measured by ground instrumentation suggests that a role could be played by the change in pixel size that occurred in August 2019 (see Section 3.1). While on one hand the S1/S4 ratio appeared more effective in detecting peaks at Stromboli when a more elongated TROPOMI product pixel was used, on the other hand the ratios using the uniform 3 × 3 and 5 × 5 windows appeared more suitable when a more squared pixel was adopted starting from August 2019.

**Figure 4:**
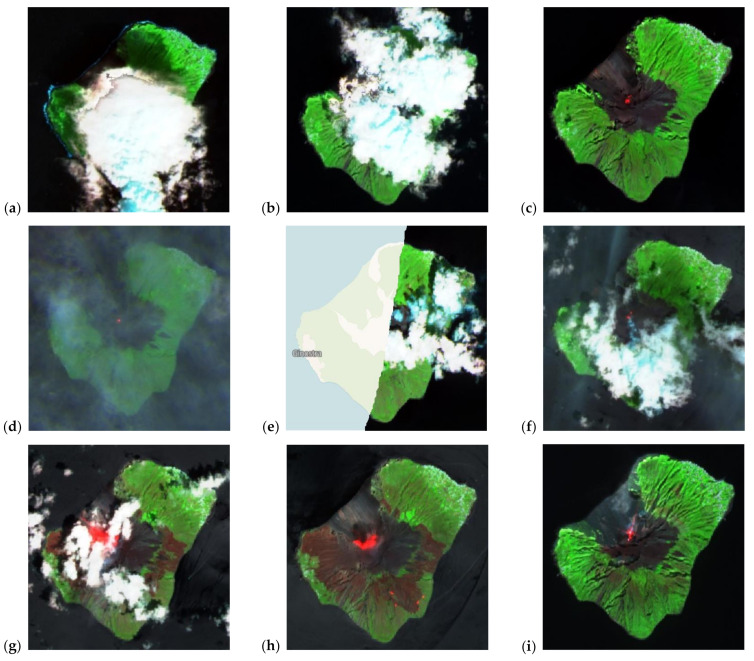
Sentinel-2 false color composites (R: band 12—short-wave infrared; G: band 8A—red edge; B: band 4—red) for: (**a**) 14 March 2019, (**b**) 19 March 2019, (**c**) 24 March 2019, (**d**) 18 May 2019, (**e**) 20 May 2019, (**f**) 23 May 2019, (**g**) 22 July 2019, (**h**) 27 July 2019 and (**i**) 6 February 2021. Contains Copernicus Sentinel-2 data 2019, processed in Sentinel Hub Playground.

In particular, if we consider the moving average in Figure 3c, S1/S4 seems to highlight the presence of SO_2_ degassing during the event period better than the other ratios. Very clear detections of activity found by using this approach are those in the period July–August 2019, when paroxysms and significant activity were also recorded by ground instrumentation (see Section 2). On 21 July 2019, the peak of S1/S4 equal to 120 indicates that the SO_2_ column density at the crater was over a hundred times higher than that found within the larger 4 × 2 window. On that day, the ROC station measured a high SO_2_ flux of 177 t/d [15]. Similarly, on 29 July and 19 August, S1/S4 ratios of 26 and around 14 were observed, respectively, and flux of 105 t/d on 29 July and 205–240 t/d on 18–20 August [15]. In all these instances, contextual Sentinel-2 data multispectral observations (i.e., acquired on the closest dates to when peaks of such ratios occur) helped to verify the presence/absence of clouds and any signs of activity at the volcano (Figure 4g,h; see also Section 4.2). From September 2019, S1/S2 and S1/S3 ratios can also be used to identify the presence of the events. For instance, on 6 February 2021, an S1/S3 ratio of about 38 matches with signs of activity at the crater, as confirmed in the Sentinel-2 image collected on the same date (see Figure 4i), though a low SO_2_ flux was recorded at the ROC station (54 t/d; [15]).

As a lesson learned from these tests, we conclude that when Sentinel-5P data are used to study SO_2_ plumes of volcanoes of “limited” areal extension, it is generally suitable to sample the single pixel (S1) and consider its value with respect to that found for larger windows. The latter, however, need to be adapted to account for the changes in the SO_2_ product pixel size to obtain the best detection rate.

### 4.2. Integrated Analysis with Sentinel-2 Observations

Integrated analysis with contextual Sentinel-2 imagery as part of a more holistic set of Copernicus Programme observations (according to the so-called “virtual constellation” concept) proves to be a multi-sensor data approach to recommend in the attempt to refine the interpretation of the findings from the Sentinel-5P SO_2_ monitoring.

A spatial analysis of the visible (bands 2–4, i.e., blue, green and red; ~490, ~560 and ~665 nm central wavelengths, respectively), red edge or narrow near-infrared (band 8A; ~865 nm) and short-wave infrared (band 12; ~2190 nm) channels can, indeed, provide helpful information to contextualize and interpret SO_2_ column density observations.

Yet, it is to be acknowledged that these satellites collect data at slightly different times of the day, or even on different dates, hence there is a temporal shift in the ground and atmosphere scenario that they observe. Nevertheless, Sentinel-2 provides a picture of the situation on the ground at a high spatial and temporal resolution (i.e., 10 m in the visible and near infrared bands; every 5 days under the same viewing conditions) that cannot be achieved with other multispectral satellites with an open data policy.

While some spurious values were filtered by the QA parameter, SO_2_ total column density peaks occurring in periods of seemingly no activity, when ground instrumentation recorded low SO_2_ flux, are still visible in the time series (Figure 2c). For instance, on 16 March 2019 (~14.4 DU, i.e., 38.6 × 10^16^ molecules/cm^2^) and 19 May 2019 (~8.1 DU, i.e., 21.7 × 10^16^ molecules/cm^2^), the ROC station recorded an SO_2_ flux of 12 and 63 t/d [15], respectively, in the NE sector of the crater terrace. The cloud flag associated with the SO_2_ product was 0.12, indicating the presence of relatively limited cloud coverage on the volcano.

On 16 March 2019, the very limited spatial coverage (only about 15%) provided by Sentinel-2 imagery at ~09:40 UTC does not allow a comprehensive assessment of the atmospheric conditions over the volcano. The presence of some clouds in the western half of the volcano is confirmed, however, by inspecting multi-spectral imagery acquired by the Visible Infrared Imaging Radiometer Suite (VIIRS) instrument onboard the Suomi National Polar-orbiting Partnership (Suomi NPP) spacecraft. The latter provides data daily with similar overpass times to Sentinel-5P (as it flies behind Suomi NPP at a distance of 3.5 min), though much lower spatial resolution (i.e., 375 m) than Sentinel-2.

On both 14 and 19 March, denser cloud cover is found over the island in Sentinel-2 imagery (Figure 4a,b), consistently with observations based on VIIRS. On those days, the cloud flag was higher (i.e., 0.47 and 0.33, respectively), though the SO_2_ data were filtered out from the series due to the additional best quality assurance thresholds used during the post-processing. This could not have been inferred based only on the QA value reported in the metadata of the TROPOMI L2 products, which was on both dates equal to 1. This confirms the need to use the additional quality filters for any SO_2_ products processed with software version v.1 (see Section 3.2) before the changes in the definition of the QA flag were implemented in version v.2 [35]. Instead, a cloud-free Sentinel-2 scene acquired a few days later, on 24 March, reveals clear signs of activity at the volcano (Figure 4c). The cloud flag of the SO_2_ product was very low, i.e., 0.05, indicating the absence of any major clouds over the island, though the SO_2_ column density was of only 1.2 DU (3.2 × 10^16^ molecules/cm^2^). On the same day, a medium level of SO_2_ flux was recorded at the ROC station, i.e., 112 t/d [15].

A similar situation happened for the days around the second SO_2_ anomaly recorded on 19 May 2019 with a QA equal to 0.9. VIIRS imagery shows dense cloud coverage over the volcano on the same day. Sentinel-2 data confirm widespread cloud cover over the eastern part of the volcano at 09:40 UTC on 20 May 2019 (Figure 4e), and the QA of the SO_2_ data for that day still indicates good quality (QA equal to 0.9). Despite the different sensing times of Sentinel-2 and Sentinel-5P over the island (and possible changes in sky conditions occurring in between the acquisitions), this check suggests that the QA control and other filtering criteria provide a good approach to ensure the best quality selection among the wealth of TROPOMI’s observations. Moreover, it is apparent that the cloud flag that is associated with SO_2_ products is an additional good indicator for the verification of cloud cover, which can be used to support data interpretation. Although ground instrumentation data indicate low SO_2_ emissions from the volcano on those dates (i.e., a few tens of t/d against hundreds of t/d during periods of high volcanic activity for Stromboli), Sentinel-2 images acquired on 18 and 23 May (Figure 4d,f) indicate the occurrence of activity at the volcano during that period and suggest that the detected SO_2_ column peak of ~8.1 DU (21.8 × 10^16^ molecules/cm^2^) on 19 May could be a reliable indication of degassing occurring on that day.

On the other hand, periods of intense volcano activity do not always associate with a strong SO_2_ emission observed by ground instrumentation. For instance, on 3 July 2019 (i.e., during the abovementioned episode that caused the death of a tourist), the recorded emissions at ROC were not particularly high, but of a medium level (SO_2_ flux of 95 t/d; [15]).

### 4.3. Conditional VCDs for Three Volcanic Scenarios

**Figure 5:**
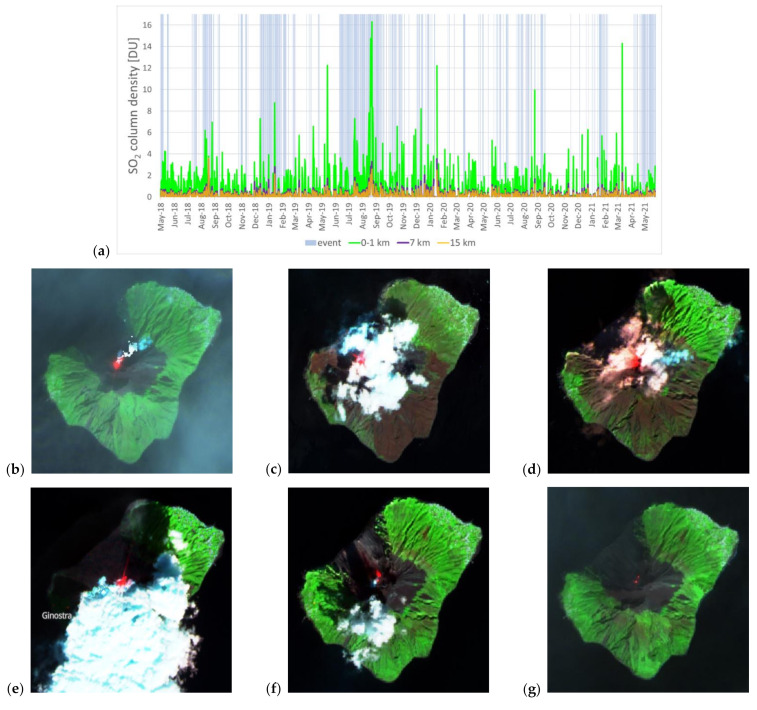
(**a**) Comparison of TROPOMI total SO_2_ vertical column density at the main crater of Stromboli for three volcanic scenarios (i.e., plume located at ground level or 7 or 15 km altitude). Sentinel-2 false color composites (R: band 12—short-wave infrared; G: band 8A—red edge; B: band 4—red) for: (**b**) 18 January 2019, (**c**) 26 August 2019, (**d**) 18 January 2020, (**e**) 23 January 2020, (**f**) 3 March 2021 and (**g**) 8 March 2021. Contains Copernicus Sentinel-2 data 2019–2021, processed in Sentinel Hub Playground.

Figure 5a shows the time series of the total SO_2_ column for the reference altitudes of 0–1, 7 and 15 km a.s.l., i.e., at the planetary boundary layer, in the free or mid-troposphere and in the lower stratosphere, respectively. These are hypothetical profiles indicating the amount of SO_2_ if it was in this layer of the atmosphere. This basically assumes three volcanic scenarios, with the plume located at (hence, the air mass factor with a presumed peak at) ground level or 7 or 15 km altitude, respectively.

Using external information on plume height (e.g., from other products, ground-based data or modeling), these profiles can be used to recalculate the SO_2_ VCD and obtain the layer of SO_2_ near a volcano. For instance Theys et al. [11] derived the plume height via triangulation from ground-based measurements, and then linearly interpolated the hypothetical profiles to match the obtained plume height.

As no information on plume height was available for the present study, the time series of the conditional VCD were inspected without further post-processing, with a view to their future exploitation and integration with data from other monitoring campaigns that might be carried out (e.g., using spectrometers onboard aircrafts).

During weak eruptions and degassing, the bulk of the SO_2_ emitted by Stromboli might be expected to be within the first few kilometers of the atmosphere. In those cases, the conditional VCD at 0–1 km (ground-level plumes) could be the series to refer to (e.g., [18]). During major explosions and paroxysms, higher plume altitudes should be accounted for (e.g., an SO_2_ plume at ~9 km altitude was reported shortly after the main explosion on 3 July 2019; [41]).

Total SO_2_ column density values of up to ~16.3 DU (43.8 × 10^16^ molecules/cm^2^) are observed from the 0–1 km altitude reference series, for instance on 28 August 2019. In contrast, the conditional value for the same day in the 7 km profile drops to ~3.3 DU (8.8 × 10^16^ molecules/cm^2^), and in the 15 km profile it drops to ~2.6 DU (7.0 × 10^16^ molecules/cm^2^). Similarly, on 20 January 2019, the values at the different reference profiles span between ~8.8 DU (23.6 × 10^16^ molecules/cm^2^) for 0–1 km, ~2.8 DU (7.6 × 10^16^ molecules/cm^2^) for 7 km and ~2.1 DU (5.7 × 10^16^ molecules/cm^2^) for 15 km. Figure 5b,c show the corresponding color composites of the Sentinel-2 multi-spectral imagery acquired on the dates closest to the dates above.

The time series in Figure 5a highlights a number of dates that were not detected as “events” by the ground-based monitoring networks. For instance, more than 12.2 DU (32.8 × 10^16^ molecules/cm^2^) were observed in the 0–1 km profile on 19 May 2019. The peak observed at this date was already analyzed in Section 4.2, in comparison with Sentinel-2 imagery (see Figure 4d–f). Similarly, VCD values of 12.2 DU (32.8 × 10^16^ molecules/cm^2^) in the 0–1 km profile were recorded on 22 January 2020, and over 5.9 DU (15.9 × 10^16^ molecules/cm^2^) on 4 March 2021. At first glance, these could appear as unrelated to Stromboli as not identified by the monitoring networks, whereas they could probably be confirmed as such via inspection of the corresponding medium resolution VIIRS multi-spectral data acquired on the same days, as well as the high resolution Sentinel-2 imagery available for the closest dates (Figure 5d–g). These images show the presence of activity at the volcano, despite the low SO_2_ flux recorded on the ground (e.g., 36, 55 and 57 t/d at ROC station on 3, 4 and 5 March 2021, respectively; [15]).

On the other hand, the peak of 14.3 DU (38.5 × 10^16^ molecules/cm^2^) recorded on 17 March 2021 in the 0–1 km profile seems rather an outlier generated by moderate cloud coverage on that day. Despite the cloud flag of ~0.08, the contextual VIIRS imagery provides evidence of clouds covering the whole volcano edifice. The SO_2_ flux recorded at the ROC station was also of 24 t/d only [15] and no evidence of activity was found from other data. Hourly wind records from the ERA5 dataset and the WMO station at Stromboli confirmed the occurrence of moderate winds (Beaufort wind force 4, i.e., 20–28 km/h) towards NW at the acquisition time of TROPOMI, likely inducing the cloud coverage over the island to change very rapidly. This could explain the inconsistency observed at this specific date. In this case, the filtering approach (as per the recommendations in [35]) did not remove this value from the series, whilst independent evidence suggests that this peak should be discarded from any further analysis.

### 4.4. Interactions between the Plumes of Stromboli and Mount Etna

**Figure 6:**
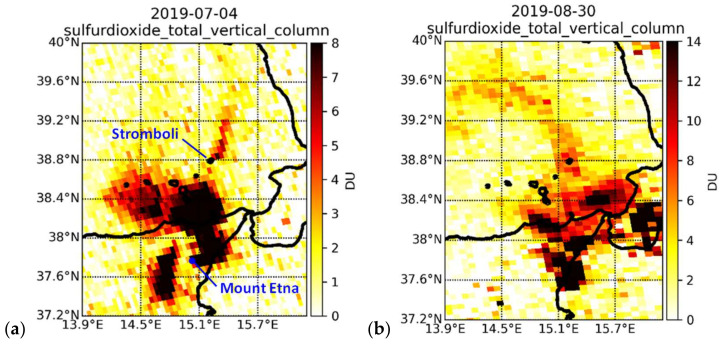
TROPOMI SO_2_ column density observations over the Aeolian archipelago and north-eastern Sicily on (**a**) 4 July 2019 at ~12:00 UTC and (**b**) 30 August 2019 at ~12:35 UTC, showing clearly distinguishable plumes of Mount Etna and Stromboli in the former, and plume overlapping by clustered degassing in the latter.

Building upon the time series analysis of the SO_2_ trend, we spatially analyzed the SO_2_ total vertical column patterns. Figure 6a shows an example where the SO_2_ volcanic plumes of Stromboli and Mount Etna are clearly visible and match with peak values in the time series extracted for the main crater of Stromboli (Figure 2d,e).

It often happens that the eruptions of the two volcanoes occur on the same days and, depending on the wind direction, it is not always possible to separate between their different emissions. As can be seen in Figure 6b, the two volcanic plumes can occasionally overlap and thus do not allow a spatial differentiation between the two emitters, or the separation between SO_2_ column density observations provided by TROPOMI.

No records on the wind direction were available at the WMO weather stations at Stromboli and Salina for either 4 July 2019 or 30 August 2019, though the SO_2_ maps suggest the presence of wind towards NE and then drifting towards NNE on 4 July (Figure 6a), and northward drifting towards NNW on 30 August (Figure 6b). At Stromboli, ERA5 hourly data confirm winds towards NE on 4 July at 11:00–13:00 UTC at 10 m height above the surface, with a Beaufort wind force of 2 (i.e., light breeze, with a speed of ~6–11 km/h). On 30 August, ERA5 data confirm the occurrence of winds with the same force directed towards N and NNE at 12:00–13:00 UTC.

Records from the WMO station at Catania (south of Mount Etna) at 12:00–13:00 UTC highlight the occurrence of ~20 km/h winds towards E and ENE on 4 July and ~17 km/h towards ESE on 30 August, explaining the local drift in the plume of Mount Etna in the proximity of the crater (e.g., at 37.8° N latitude in Figure 6a).

The plume overlapping by clustered degassing volcanoes (within ~50 km) is a delicate problem to consider in satellite studies of volcanic emissions, and could bring a data value overestimation [42,43]. While most of the literature was based on predecessor satellites, even if TROPOMI helps to reduce these biases and improve the overall quality of the data [18,23], its products still suffer from the potential occurrence of this type of interference.

Therefore, in our approach, we address this issue by comparing data collected in the days before and after the occurrence of the spatial overlap and via analysis of time lapses (see Section 4.5). An estimation of the VCD and emissions for the day affected by the overlapping could be provided via regression on the values observed of preceding and following days, and also with the help of the ground instruments.

### 4.5. Time Lapse of the 2019–2021 Eruptions

**Figure 7:**
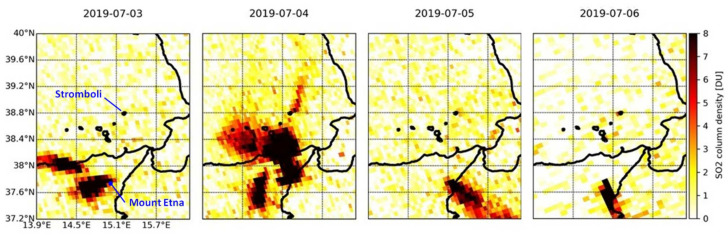
Time lapse of TROPOMI SO_2_ column density observations over the Aeolian archipelago and north-eastern Sicily in the period 3–6 July 2019.

**Figure 8:**
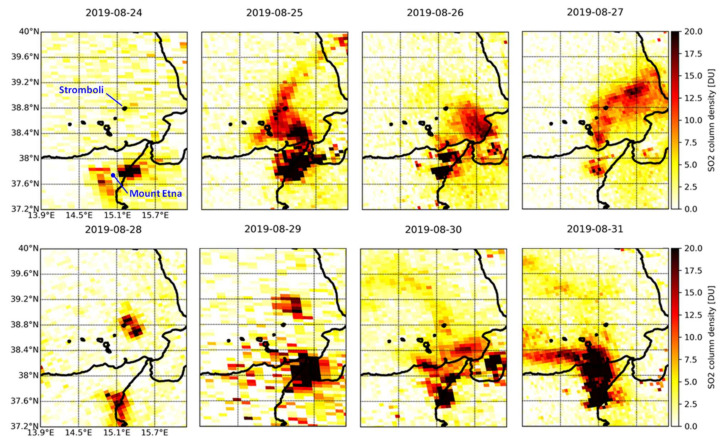
Time lapse of TROPOMI SO_2_ column density observations over the Aeolian archipelago and north-eastern Sicily in the period 24–31 August 2019.

**Figure 9:**
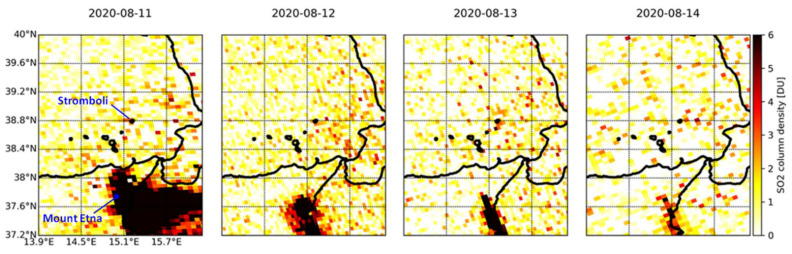
Time lapse of TROPOMI SO_2_ column density observations over the Aeolian archipelago and north-eastern Sicily in the period 11–14 August 2020.

**Figure 10:**
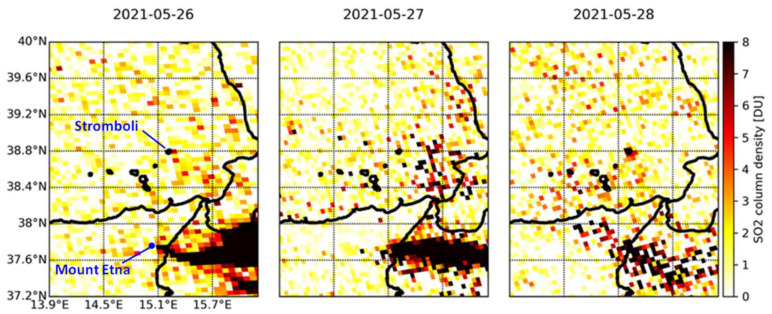
Time lapse of TROPOMI SO_2_ column density observations over the Aeolian archipelago and north-eastern Sicily in the period 26–28 May 2021.

Based on the history of recent eruptions (Section 2), four major events were selected and their evolution is shown through daily time lapses of the total SO_2_ column:

- The period of activity that started on 3 July 2019 with a paroxysm (Figure 7);
- A further paroxysm that occurred on 28 August 2019 (Figure 8), followed by two new bursts of slightly lower intensity between 29 and 30 August 2019:
- The major explosions that occurred on 13 August 2020 (Figure 9);
- A more recent period of activity that started on 19 May 2021 and lasted for several days (Figure 10).

The situation is highlighted, day by day, by the changing peak value of the detected total vertical column of SO_2_ in each of these time lapses.

Within the first time lapse for 3–6 July 2019 (Figure 7), as it can be seen from the maps, the day of greatest activity during the first period was on 4 July (SO_2_ flux of 80 t/d measured at the ROC station; [15]), when the plumes of both Stromboli and Mount Etna could be observed and distinguished, with ~1.9 DU (5.1 × 10^16^ molecules/cm^2^) over Stromboli, and a maximum of more than 10 DU (26.9 × 10^16^ molecules/cm^2^) just off the northern coastline of Sicily, clearly generated from Mount Etna. In this case, the two eruptions stand out perfectly and can be easily discerned one from each other. On 5–6 July, Mount Etna’s plume drifted towards the SE and SSE, hence away from Stromboli (Figure 7).

This is not the case for the second time lapse at the end of August 2019 (Figure 8), when the eruption of Mount Etna occurred contemporarily with that of Stromboli and, due to the winds, the bigger plume of Mount Etna covered Stromboli for several days, e.g., on 25–27 August. During those three days, the two plumes cannot be easily separated from each other in the maps of the time lapse, and the observed SO_2_ column values (Figure 2d,e) cannot be robustly associated with emissions from Stromboli only. Indeed, many peaks that are identified in the time series actually could be mainly due to Mount Etna’s emissions (e.g., the peak of around 13 DU observed on 25 August over Stromboli). However, the recorded high values of SO_2_ with respect to periods of no activity (or low emissions) are still worth considering, even if they are associated with clustered signals. The northward SO_2_ plume direction that can be observed over Stromboli in the time lapse on 25 August at ~12:25 UTC is consistent with the direction indicated by ERA5 hourly data on the same day at 12:00–13:00 UTC.

From the time lapse, however, it is also clear that on 28 August at ~11:30 UTC the two signals did not overlap. This also matches with what the Copernicus Emergency Management Service has documented [44]. Therefore, for that day it is possible to carry out an analysis of the emissions from Stromboli, without interference from Mount Etna.

A peak of ~3.0 DU (8.1 × 10^16^ molecules/cm^2^) was detected at the main crater of Stromboli, and 22.1 DU (59.5 × 10^16^ molecules/cm^2^) a few kilometers to its northeast. This is consistent with ERA5 historical data that show the occurrence of winds towards ENE on 28 August at 11:00–12:00 UTC, suggesting a drift towards NE of the SO_2_ plume emitted by Stromboli. At the ROC station, a very high SO_2_ flux of 261 t/d was recorded on 28 August, consistently with the same range or even higher values on the previous and following days, e.g., 306 t/d (very high) on 24 August and 200–230 t/d (high) on 25–27 and 29 August [15]. This circumstance highlights how the temporal trend and spatial pattern analysis are mutually complementary to achieve a comprehensive understanding of the emissions.

On the other side, Figure 2d,e reveals peaks outside periods of activity for Stromboli. This can be explained by the direction of the winds above Mount Etna, which brings its plume towards Stromboli and sometimes makes it cover the island, as if it were Stromboli itself in the degassing or the eruption phase. As a lesson learned, in future observation campaigns, it will be necessary to take into account these possible interferences. These can largely impact the SO_2_ column density values detected at Stromboli, and thus their analysis and interpretation, in the framework of monitoring volcanic activity.

The SO_2_ time lapse for the period 11–14 August 2020 (Figure 9) shows a much less distinguishable signal over Stromboli, with a column density of ~2.6 DU (7.1 × 10^16^ molecules/cm^2^) on 13 August at the main crater, though without a clearly detectable plume, in contrast with that of Mount Etna that extends for several square kilometers towards the east and south of its crater. The SO_2_ flux recorded on the ground at the ROC station was 59 t/d (low) on 11 August, and 112, 99 and 103 t/d (medium) on 12, 13 and 14 August, respectively [15].

After months with low activity, a little bit more visible plume is found over Stromboli on 28 May 2021 with a column density of ~5.6 DU (15.2 × 10^16^ molecules/cm^2^), as it can be observed from the time lapse in Figure 10. The winds recorded at 10:00–13:00 UTC on that day at the WMO weather stations in Stromboli and Salina were relatively slow (~5 km/h). Additionally, in this case there is a difference between Stromboli and Mount Etna’s emission and respective plumes, with that of Mount Etna traveling towards the east and not overlapping with Stromboli. During these days, the SO_2_ flux recorded at the ROC station was 123, 166 and 76 t/d (medium) on 26, 27 and 28 May, respectively [15].

## 5. Conclusions

The investigation of SO_2_ emissions at the Stromboli volcano using the full dataset of TROPOMI SO_2_ VCD time series collected from 6 May 2018 to 31 May 2021 proves that Copernicus Sentinel-5P is a valuable space asset for monitoring the volcanic activity of small-size Strombolian volcanoes, characterized by violent and explosive eruptions, as well as by persistent degassing. While an increasing body of literature is being published on the use of these data for volcanic studies, the present research aims to deepen the discussion on the practical technical issues involved in the handling and post-processing of these geophysical data that are yet to become of common and standard use across the scientific community interested in volcanological applications.

The methodological workflow that is proposed in this paper encompasses an ad hoc step of spatial sampling that is aimed to check the correctness of the location from which SO_2_ VCD values are extracted with regard to the volcano edifice size and its orientation compared to the TROPOMI pixel size and extent. Our tests suggest that in the case of volcanoes of “limited” areal extension, i.e., comparable to the pixel size of TROPOMI data, it is generally suitable to sample the single pixel and consider its value with respect to that found for larger averaging windows.

Another important aspect is to implement data filtering by quality and outlier removal that not only comply with the recommendations issued by the Mission Performance Centre calibration/validation experts and the Sentinel-5P Validation Team, but also account for the specific characteristics of the studied volcano. In the case of Stromboli, the QA threshold applied to remove low quality data was set to 0.5, and the outlier filtering threshold to exclude negative SO_2_ column values was set at −0.001 mol./m^2^. Additional stringent criteria on the SO_2_ total air mass, cloud fraction, solar zenith angle and presence of snow/ice were also used to achieve best quality data selection.

The time series of total SO_2_ column density for the hypothetical profile at the 0–1 km peak altitude was the profile used for reference, as the bulk of the SO_2_ emitted by Stromboli is expected to be within the first kilometer of the atmosphere (i.e., ground-level plume and degassing). During major explosions and paroxysms, as well as on some days of moderate activity at the volcano, the data showed very clearly a series of significant total VCD at the main crater.

Time series analysis at Stromboli suggests that SO_2_ total VCD peaks as captured by TROPOMI data can serve as reliable proxies of volcanic activity and, as such, can be used to investigate other volcanoes when information about events is scarce or absent. However, it is not always trivial to associate SO_2_ total VCD peaks with moderate to very high SO_2_ emissions recorded by ground-sensor data, and reported in volcanological observatories bulletins. In this regard, the inspection of contextual Sentinel-2 multispectral observations in the visible, near and short-wave infrared (as well as Suomi NPP VIIRS data) provides an effective means to refine the interpretation of SO_2_ VCD peaks when they occur outside known periods of significant emissions. Although some limitations may occasionally constrain the analysis (i.e., cloud coverage that may hinder the visibility; time lag between the acquisition date of Sentinel-2 vs. TROPOMI data), this multi-sensor data approach adheres to the holistic concept of multi-band and multi-mission observations that are behind the whole Copernicus Earth Observation Programme.

Finally, the multi-temporal analysis of daily time lapses of SO_2_ VCD during the paroxysms that occurred in July–August 2019, major explosions in August 2020 and a more recent period of activity in May 2021 demonstrates that the proposed approach is successful in showing the SO_2_ degassing associated with these events, and warning whenever the total SO_2_ column density values at Stromboli may be overestimated due to clustering with the plume of the Mount Etna volcano. This geographical parameter, alongside wind direction, has to be accounted for whenever the nearly point-wise volcanic source under investigation is located in proximity to other SO_2_ emission sources, either natural (e.g., Mount Etna) or anthropogenic, that may interfere.

## Figures and Tables

**Table 1:** Total number of days during the May 2018–May 2021 period when the ratios between observations of total SO_2_ VCD at the single pixel (S1) and within the averaging windows (S2: 3 × 3, S3: 5 × 5 and S4: 4 × 2) exceeded the selected thresholds. Such occurrences are then distinguished according to the number of days when low (<70 t/d) and medium to very high (≥70 t/d) SO_2_ flux was detected at ROC station.

Ratio	Condition	No. of Occurrences	SO_2_ Flux at ROC Station	
			≥70 t/d	<70 t/d
S1/S2	5 ≤ S1/S2 < 10	24	5	19
	S1/S2 ≥ 10	14	4	6
S1/S3	5 ≤ S1/S3 < 10	45	12	32
	S1/S3 ≥ 10	29	6	20
S1/S4	5 ≤ S1/S4 < 10	37	12	21
	S1/S4 ≥ 10	22	9	9

## Data Availability

Sentinel-5P TROPOMI and Sentinel-2 MSI data are made available by ESA in the Copernicus Open Access Hub. Suomi NPP VIIRS imagery can be consulted using NASA’s Earth Observing System Data and Information System (EOSDIS). WMO wind records are made available from Meteostat. ERA5 hourly data are distributed by ECMWF via the Copernicus Climate Change Service (C3S).
